# Role of Risk Factors in Developing Osmotic Demyelination Syndrome During Correction of Hyponatremia: A Case Study

**DOI:** 10.7759/cureus.6547

**Published:** 2020-01-02

**Authors:** Tom D.Y. Reijnders, Wilbert M.T. Janssen, S.M. Laila Niamut, Andrea B Kramer

**Affiliations:** 1 Center for Experimental and Molecular Medicine, Amsterdam University Medical Centers, Location Academic Medical Center, Amsterdam, NLD; 2 Internal Medicine, Martini Hospital, Groningen, NLD

**Keywords:** hyponatremia, osmotic demyelination syndrome, central pontine myelinolysis, sodium, risk factors, complication, alcoholism, malnutrition

## Abstract

This case report describes a 57-year-old man who presented first with lethargy and dysarthria due to hyponatremia resulting from poor intake and diuretics. One week after discharge, he returned with confusion, ataxia and dysphagia, and he ultimately turned out to have developed an osmotic demyelination syndrome (ODS). In his first hospital admission, his serum sodium was corrected without new neurological symptoms occurring. In retrospect, he had several risk factors for the development of ODS during the correction of hyponatremia. The serum sodium correction rate only briefly exceeded the recommended limits. This case underlines that (1) extra awareness of the serum sodium correction rate is warranted in patients with risk factors, (2) factors other than sodium can play an important role in the development of ODS and (3) that the manifestations of ODS can be delayed substantially after an incident of osmotic stress.

## Introduction

Severe hyponatremia is strongly associated with increased mortality and should therefore always be treated [1,2]. Correction of the serum sodium can result in an osmotic demyelination syndrome (ODS), a severe and potentially untreatable neurological condition [3]. This case report describes two subsequent admissions of a patient who was initially thought to be delirious but turned out to have developed ODS. We focus on the risk factors that - despite careful correction of hyponatremia - can nevertheless lead to severe complications.

## Case presentation

The first admission

A 57-year-old man, with a history of hypertension, diabetes mellitus type 2, gout and chronic alcohol abuse, presented to the emergency department with lethargy, dysarthria and lack of coordination. He had been passing loose stools recently. His outpatient medications consisted of rosuvastatin, metformin, enalapril, amlodipine and hydrochlorothiazide. The patient was not evidently dehydrated on physical examination. Laboratory tests revealed hyponatremia (105 mmol/L) with low urine sodium and acute kidney injury (Table 1). Potassium and glucose were within normal range.

**Table 1:** Relevant lab test results at presentation (first admission)

	Value (reference range)	
Serum		
Sodium	105	mmol/L (135-145)
Potassium	4.5	mmol/L (3.5-5.0)
Creatinine	147	µmol/L (50-100)
	1.7	mg/dL (0.6-1.1)
Urea	15.3	mmol/L (2.5-7.5)
	91.9	mg/dL (15.0-45.0)
Glucose	6.7	mmol/L (4.0-7.8)
	120.7	mg/dL (72.1-140.5)
Urine		
Sodium	<20	mmol/L
Osmolality	224	mOsm/kg (300-900)

**Figure 1:**
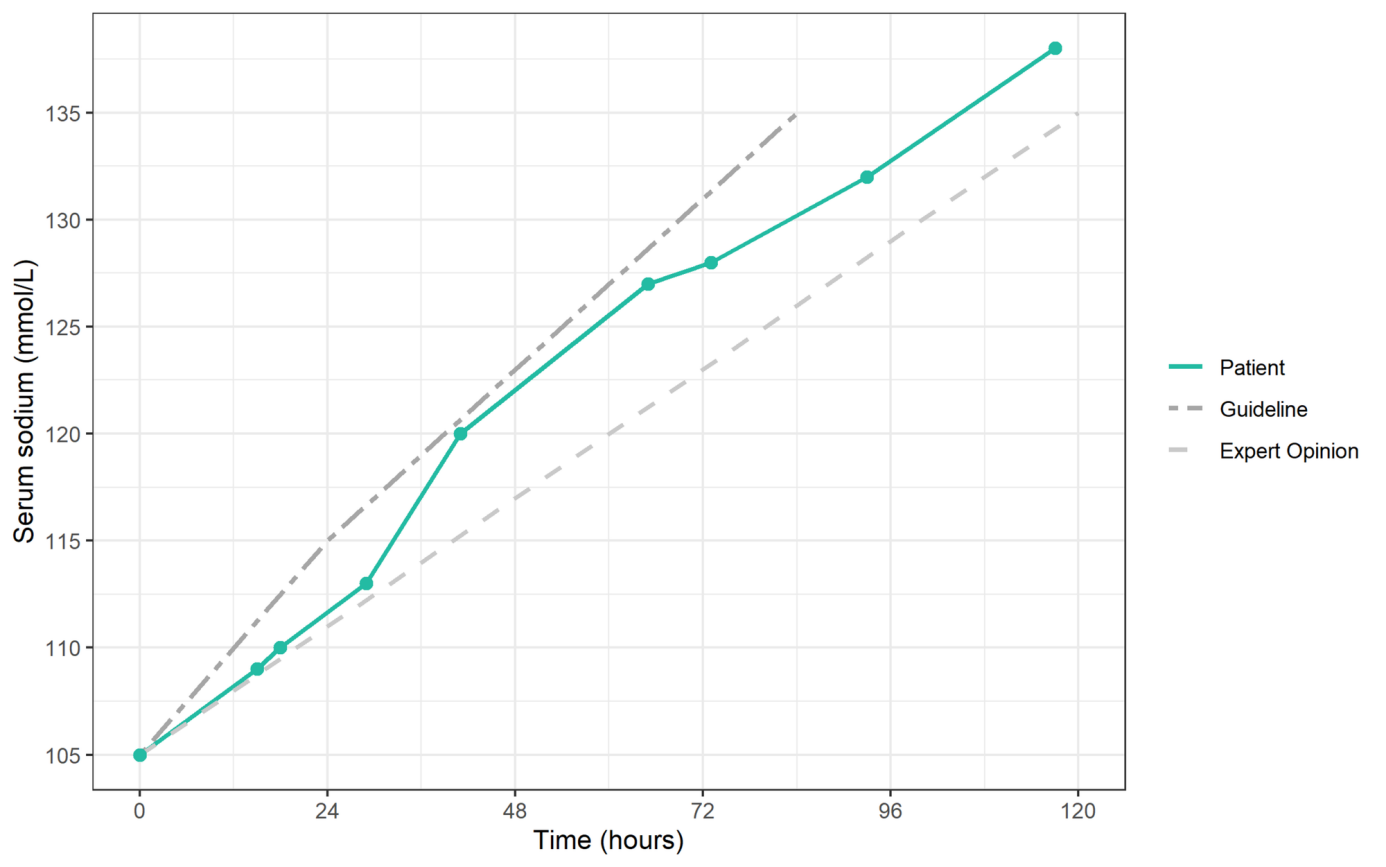
Course of the correction of hyponatremia Graph depicting the correction of hyponatremia in our patient (● is a point of measurement) compared with the European Guidelines (a maximum of 10 mmol/L in the first 24 hours, followed by a maximum of 8 mmol/L in the subsequent days) [4] and the ‘expert opinion’ (a goal of 4-6 mmol/L/day) [5].

At this point, we considered the symptoms to fit with symptomatic hyponatremia due to poor intake and hydrochlorothiazide use. We started an isotonic saline infusion and discontinued the hydrochlorothiazide. Serum sodium increased gradually (Figure 1); we adjusted the infusion rate in response to fluctuations in the correction speed according to the European guideline [4]. The acute kidney injury restored fully.

**Figure 2:**
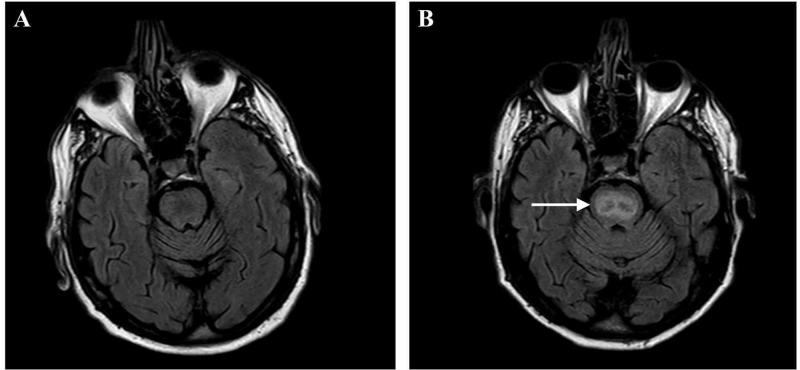
T2-weighted axial brain MRI of our patient (A) The first MRI, made during the first admission four days after the correction of hyponatremia, in which no significant abnormalities were observed. (B) The second MRI, made more than three weeks after the first MRI towards the end of the second admission, in which a clearly increased signal intensity is visible in the pons (denoted by →), characteristic for an osmotic demyelination syndrome.

During admission, the patient became increasingly confused, which we deemed to be an alcohol withdrawal delirium after ceasing to drink three to four beers per day. We treated the delirium with haloperidol and lorazepam. The neurologist’s examination revealed no abnormalities, aside from a slight tremor. Magnetic resonance imaging (MRI) of the brain showed no significant abnormalities (Figure 2A). Four days after attaining a serum sodium within the normal range, the symptoms of the delirium had improved substantially, and the patient was self-sufficient in activities of daily living. He was discharged with a three-day supply of haloperidol and lorazepam.

The second admission

The patient returned to the emergency department one week later. His confusion had worsened substantially since discontinuing the medication; he was agitated and hallucinating. He now required assistance with all activities of daily living. However, he had not consumed alcohol since before the previous admission.

The patient appeared apathetic, responded incoherently to questions and sometimes spoke unintelligibly. His vital parameters were normal, and the laboratory tests were unremarkable aside from an isolated elevation of gamma-glutamyl transferase of 201 (<55 U/L). His serum sodium was 143 mmol/L at this point. Under the preliminary diagnosis of persistent delirium resulting from premature discontinuation of haloperidol and lorazepam, we restarted haloperidol and lorazepam and admitted the patient for observation.

The patient deteriorated despite the medication. He fell twice during the night and developed myoclonus and dysphagia. Contrary to the first admission, neurological symptoms were clearly present at this point: ataxia, an abnormal plantar reflex (Babinski sign), dysarthria and possibly aphasia, indicating a disorder in the central nervous system. Because of the preceding severe hyponatremia and alcohol abuse, our differential diagnosis now included ODS and Wernicke’s encephalopathy. We excluded the latter when several days of high-dose intravenous thiamine failed to improve the neurological symptoms.

A few days later, the patient developed an aspiration pneumonia due to dysphagia, and he had to be transferred to an intensive care unit (ICU).

A second MRI of the brain (Figure 2B) was performed to look for ODS. In stark contrast with the first MRI, the second MRI revealed a markedly increased signal intensity in the pons characteristic for ODS. When the patient was ready for discharge, his dysarthria and dysphagia had improved substantially. He was transferred to a rehabilitation facility for further recovery. 

## Discussion

Hyponatremia

Hyponatremia is the most common electrolyte disorder with an estimated prevalence of 1.72% in the general population (in the United States) and a clear association with increased mortality [1,2]. Most symptoms in acute and severe hyponatremia occur due to cerebral edema resulting from hypo-osmolality [6]. In acute hyponatremia (e.g., marathon runners that drink a lot of water and simultaneously lose sodium through sweating), patients suffer headaches, malaise, seizures or even coma. In chronic hyponatremia, symptoms are subtler and include fatigue, muscle spasms and cognitive decline. However, chronic hyponatremia is oftentimes asymptomatic.

The urine osmolality, urine sodium and the volume status of the patient can aid in assessing the cause of hyponatremia [4]. In this case, several factors influence both the osmolality of the serum and the interstitial fluid: thiazide diuretics can induce severe hyponatremia [7]; malnutrition and loose stools can lead to sodium deficiency; excessive consumption of hypotonic fluids such as beer (beer potomania) - especially when combined with insufficient intake of protein and sodium (tea and toast) - can reduce the renal free water clearance [8]. While thiazide-induced hyponatremia commonly presents with higher urine sodium [4], the low urine sodium likely resulted from the other factors and the prerenal acute kidney injury, perhaps in tandem with poor adherence to the diuretic prior to presentation due to confusion. The next sections will discuss the treatment of these causes of hyponatremia and elucidate their role as risk factors in the overcorrection of serum sodium and the development of ODS.

Guidelines for treatment 

Depending on the severity and cause of hyponatremia, the treatment consists of (a combination of) hypertonic saline, isotonic saline or fluid restriction [5]. The goal is a gradual and controlled increase in serum sodium to achieve normal levels. The European guidelines recommend a maximum increase of 10 mmol/L in the first 24 hours, followed by a maximum increase of 8 mmol/L the following days [4]. Severe symptoms justify rapid initial correction - 5 mmol/L in one hour, followed by 1 mmol/L per hour if symptoms persist - provided that one stays within the limit of 10 mmol/L in the first 24 hours. However, because estimating the increase in serum sodium is notoriously difficult and the risk of overcorrection is substantial, some experts recommend a more conservative target of 4-6 mmol/L per day [5].

Figure 1 shows the course of the serum sodium for our patient compared with the European guidelines, as well as with the stricter ‘expert opinion’ [4,5]. The administration of hypertonic saline was not common practice in our hospital at the time of this case. In our patient, the correction speed staid around 6 mmol/L/day during the first 29 hours with a rise in serum sodium from 105 to 113 mmol/L. Starting from 110 mmol/L, with a consistent saline infusion rate, the serum sodium increased by three points in about 12 hours, followed by a sudden jump of 7 mmol/L in the next 12 hours. The following 24 hours, despite discontinuing the saline infusion for more than half the day, the serum sodium increased by another 7 to 127 mmol/L. This course emphasizes the unpredictable nature of changes in serum sodium and the need to check the levels frequently. The guidelines corroborate this unpredictability and provide strategies to respond to overcorrection [4,5].

Saline infusion and stopping hydrochlorothiazide are the basis for serum sodium to rise. A continuous saline infusion may cause a sudden increase in serum sodium upon restoring fluid depletion, because the stimulus for antidiuretic hormone-mediated free water retention is eliminated at that point. This may help explain the sudden jump in serum sodium in our patient. However, other factors may also contribute to a rise in serum sodium and may, apart or in combination, lead to overcorrection of serum sodium, despite calculating infusions according to guidelines (Table 2).

**Table 2:** Risk factors for overcorrection of serum sodium and development of ODS ODS, osmotic demyelination syndrome; ADH, antidiuretic hormone; SIADH, syndrome of inappropriate ADH secretion.

Risk factors for overcorrection of serum sodium
Elimination of a cause of SIADH (medication, infection, etc.)
Discontinuation of ADH agonists or treatment with ADH antagonists
Administration of corticosteroids in hyponatremia due to adrenal insufficiency
Discontinuation of thiazide diuretics
Administration of normal saline in fluid depletion
Concurrent correction of hypokalemia
High protein intake in patients with malnutrition
Risk factors for ODS
Severe chronic hyponatremia (<120 mmol/L)
(Too rapid) correction of serum sodium and other forms of osmotic stress
Hypokalemia (irrespective of correction speed)
Chronic alcohol abuse
Malnutrition
Liver disease and liver transplantation

For instance, concurrent correction of the effective osmole potassium is an important risk factor for overcorrection: administered potassium ions rapidly enter the cells in exchange for sodium ions, which causes extracellular sodium to rise [9]. Our patient’s potassium remained stable around 4 mmol/L during the first admission, and there was no need for potassium infusion.

The degree to which abstaining from alcohol and the associated tubular dysfunctions may play a role in overcorrection is uncertain. Alcohol appears to be an underestimated factor: a large proportion of reported cases of ODS are associated with alcohol abuse [4]. Both acute and chronic alcohol abuse can directly (and indirectly through effects on the liver) disturb the homeostasis of fluid and various electrolytes, although it is hard to estimate to which extent these factors contributed in our patient after ceasing to drink beer [10].

Finally, protein intake leads to an increased free water clearance in the kidney and consequently a rise in serum sodium. Some authors therefore recommend abstaining from oral intake in the first 24 hours of correction of hyponatremia and to use dextrose 5% in water (D5W) infusion when calories are essential [11]. Moreover, it is conceivable that protein intake not only leads to increased levels of protein degradation products in the kidneys, but also in the brain interstitial space, where it may contribute to the development of ODS by itself. Our patient was offered a standard diet.

Osmotic demyelination syndrome

During chronic hyponatremia, brain cells gradually attain a new osmotic balance with the interstitial space and thereby limit cerebral edema [3]. In this state, brain cells are highly sensitive to rapid changes in concentrations of effective osmoles. This osmotic stress, such as a rapid increase in serum sodium or accumulation of protein degradation products, can lead to apoptosis of astrocytes in the central nervous system. This process culminates in ODS [3,12]. Alcohol abuse and malnutrition exacerbate the problem through deficiency of energy and nutrients [13]. In these situations, ODS can occur with correction speeds below 0.5 mmol/L/hour (12 mmol/L/day) [14]. Thus, correction speeds should not exceed 4-6 mmol/L/day when these risk factors are present, and prudence with meals rich in protein is likely also warranted.

ODS has also been linked to other forms of osmotic stress, such as end-stage renal disease, hypophosphatemia and hyperosmolar hyperglycemia [15]. Osmotic stress should therefore always be avoided, especially in the context of chronic and severe hyponatremia combined with other risk factors for ODS (Table 2). A slow and controlled correction of chronic hyponatremia can alleviate its symptoms while minimizing the risk of excessive osmotic stress. Rapid correction of hyponatremia is mandatory only when it is severely symptomatic.

The symptoms of ODS generally start several days after an incident of osmotic stress. In patients with chronic alcohol abuse, cerebellar symptoms - such as ataxia, dysarthria and tremors - seem to predominate [16]. Other symptoms include dysphagia, paresis, altered mental state and seizures.

The diagnosis of ODS is made by brain MRI. It is important to realize that it can take several weeks after the symptoms of ODS start before the characteristic signs of pontine or extrapontine demyelination are visible [13]. The first brain MRI of our patient showed no abnormalities (Figure 2A). The second MRI, more than three weeks later, showed a distinctly altered pontine signal intensity (without extrapontine involvement), characteristic for ODS (Figure 2B).

No treatment exists when ODS has definitively occurred. However, there are indications from case reports and animal studies that lowering the serum sodium directly after the appearance of symptoms can reverse the disease [17,18]. The first symptoms of our patient likely started at home, which rendered this strategy impossible.

The prognosis of ODS is poor but variable. A study of 36 patients with ODS in an ICU reported an unfavorable outcome after one year (death or disability) in 50% of patients. However, of the 25 patients that survived the hospital admission, 56% had no or minimal limitations in daily activities after one year [19]. The symptoms of our patient had improved substantially upon transfer to the rehabilitation facility.

## Conclusions

Hyponatremia is the most common electrolyte disorder and is strongly associated with increased mortality. A gradual and controlled correction of serum sodium is crucial to prevent complications. This case illustrates that even when serum sodium is corrected according to the guidelines, a patient with risk factors - such as alcohol abuse and malnutrition - can develop an ODS. Thus, extra awareness of the serum sodium correction rate is warranted in patients with risk factors, since factors other than sodium can play an important role in the development of ODS. The clinical and especially the radiological manifestations of ODS can be delayed substantially after an incident of osmotic stress.
